# Are adult cystic fibrosis patients satisfied with medication treatment?

**DOI:** 10.1186/s13023-025-03676-6

**Published:** 2025-04-12

**Authors:** Esen Deniz Akman, Nadir Yalçın, Oğuz Karcıoğlu, Ebru Damadoğlu, Ali Fuat Kalyoncu, Kutay Demirkan

**Affiliations:** 1 Department of Clinical Pharmacy, Faculty of Pharmacy, Hacettepe University, 06230 Ankara, Türkiye; 2 Department of Pulmonary Medicine, Faculty of Medicine, Hacettepe University, 06230 Ankara, Türkiye

**Keywords:** Cystic fibrosis, Adults, Treatment satisfaction, Drug related problems, Modulators

## Abstract

**Background:**

Treatment satisfaction can be described as the patient’s experience in patients with cystic fibrosis (CF). It can be influenced using modulators and clinical characteristics. The aims of this study were to evaluate and compare adult CF patients with and without modulators regarding treatment satisfaction, related factors and to manage their drug related problems (DRPs).

**Methods:**

A single-center prospective cohort study was conducted between June 2023 and January 2024. Treatment Satisfaction Questionnaire for Medication (TSQM 1.4), including effectiveness, side effects, convenience, global satisfaction domains, CF Questionnaire-Revised (CFQ-R), and Medication Adherence Report Scale were applied and assessed with and without modulator therapy groups. The relationship between clinical characteristics and TSQM was analyzed by correlations and regression analysis. Recommendations on DRPs identified by clinical pharmacists were made to the physicians and patients and classified according to Pharmaceutical Care Network Europe (PCNE v9.1).

**Results:**

A total of 110 patients with 51 modulator therapy and 59 without modulator therapy were included. The mean global satisfaction score of modulator users was found to be 19.733 (*p* < 0.001) points higher than non-users. When the CFQ-R treatment burden score improved by 1point, global satisfaction score increased by 0.233 points (*p* < 0.001). When the number of hospitalizations increased by 1 day, the global satisfaction score decreased by 4.751 points (*p* < 0.001). A total of 84 DRPs were identified, and 69 (82.1%) of them were resolved.

**Conclusions:**

Treatment satisfaction in adult CF patients is influenced by modulators, treatment burden, and clinical factors, so access to modulators is important. This is the first study to classify DRPs according to PCNE in CF. Clinical pharmacists contribute to the management of CF.

## Introduction

Cystic fibrosis (CF) is a rare genetic disease in which mutations in the gene encoding for the CF transmembrane conductance regulator protein result in a multisystem disease. Although CF has been recognized as a pediatric disease for many years, the progress in treatment options has led to an increase in life expectancy [1]. Until a few years ago, available treatments only aimed to alleviate the symptoms caused by the disease, but recently modulator therapies have been developed that minimize most symptoms, improve patients’ quality of life, and lead to a reduction or discontinuation of symptomatic treatment [2]. Treatment satisfaction can be defined as the patient’s evaluation of the process of taking the medication, the short-term effects of the medication, and the associated long-term outcomes [3]. Significant relationships were found between adherence with treatment, quality of life, clinical status, and treatment satisfaction in various diseases [4, 5]. Considering the treatment burden of patients, drug costs, and the use of a large number of devices, treatment satisfaction may be affected in CF. Especially with the use of modulator therapies, treatment satisfaction may be affected by related factors. Treatment satisfaction in CF patients was analyzed only for inhaled tobramycin treatment, however other treatments were not evaluated [6]. As CF patients live with a high treatment burden, it is believed that understanding the satisfaction with treatment in this disease group and the quality of life, adherence to treatment and clinical parameters that may be related to it, and eliminating the problems that may be related to medications on these parameters, will benefit the individual treatment management of these patients. Due to the complexity of medication management in CF, the Cystic Fibrosis Foundation (CFF) recommends a multidisciplinary team approach to the care of CF patients. The CFF guidelines recommend that pharmacists should be members of the multidisciplinary CF care team, including pulmonologists, gastroenterologists, dietitians, and nurses and be involved in the development of services provided to patients [7]. The primary aim of this study was to evaluate treatment satisfaction and related factors in CF patients aged 18 years and older who were followed up in the pulmonary outpatient clinic. The secondary aims were to compare treatment satisfaction and related factors in CF patients with and without modulator therapy and to manage drug-related problems in all patients.

## Methods

### Study design

This prospective single-center cohort study was conducted between June 2023 and January 2024. Patients with CF over 18 years of age who were followed in the pulmonary outpatient clinic of a tertiary care university hospital were included in order to evaluate their treatment satisfaction and related factors. Patients with pulmonary exacerbation, hospitalization, alteration in medication, no medication usage in the last month, impaired cognitive function, difficulty in communication, history of lung or liver transplantation, pregnants and patients who disagreed to participate in the study were excluded. Patients were also evaluated in two groups as modulator and non-modulator groups. The management of drug-related problems was assessed in all patients. The study was approved by the local ethics committee (decision no: GO 23/636, date: 25 July 2023).

### Data collection

Data were prospectively collected by a clinical pharmacist in a pulmonary medicine outpatient clinic where the largest number of adults with CF are followed up nationally.

Treatment Satisfaction Questionnaire for Medication (TSQM 1.4) was used to assess patients’ treatment satisfaction for medication TSQM 1.4 consists of 14 questions and has domains on effectiveness, side effects, convenience, and global satisfaction. The higher the score ranging from 0 to 100 in each domains of the questionnaire, the higher the level of satisfaction in those domains [8].

The quality of life was evaluated with the Cystic Fibrosis Questionnaire-Revised (CFQ-R) adult version. This questionnaire consists of a total of 50 questions and 12 sections (physical, vitality, emotional, nutrition, treatment burden, health, social, body, role, weight, respiratory, and digestion). Each domain is scored between 0 and 100. As the score increases, the quality of life increases for each domain [9, 10].

The Medication Adherence Report Scale (MARS) was used to evaluate the medication adherence of the patients. The scale is a 5-point Likert scale (Always = 1, Frequently = 2, Sometimes = 3, Rarely = 4, Never = 5) and consists of 5 questions. Each question is scored from 1 to 5, so the total score is in the range 5–25 points. The higher the score, the higher the adherence to medical treatment [11].

TSQM 1.4 and MARS were applied face-to-face in the outpatient clinic. As adult CF patients were relatively young and proficient in computer use, the CFQ-R was sent by email to them because it was time consuming for the patients.

Patients’ medications were systematically evaluated by the clinical pharmacist in terms of potentially interferes. Patients were informed about their treatments, especially on modulators. When drug-related problems (DRPs) were detected and checked by Lexicomp UpToDate® [12], CFF guidelines [13], European Cystic Fibrosis Society (ECFS) guidelines [14], and ESPEN-ESPHGAN-ECFS 2016 Cystic Fibrosis Guideline [15], recommendations were made to the physician for the management of DRPs. DRPs were classified using the Pharmaceutical Care Network Europe (PCNE) 9.1, which has 3 primary fields for problems, 9 primary fields for causes, 5 primary fields for planned interventions, 3 primary fields for acceptance level (of interventions), and 4 primary fields for status of the problem. Problems include treatment effectiveness and safety, while reasons include drug selection, dose selection, and treatment duration [16].

### Statistical analysis

Sample size calculation was performed with the G-Power 3.0.10 program. The results obtained and the demographic characteristics and clinical data of the patients were analyzed using the SPSS Version 23.0 statistical analysis program.

As descriptive statistics, mean and standard deviation (SD) or median and minimum–maximum values for numerical variables and number and percentage values for categorical variables were given. The normality assumption was analyzed by Kolmogorov–Smirnov test. In the comparison of numerical data, Mann–Whitney U test was used for data that did not show normal distribution. Pearson Chi-Square or Fisher’s Exact test was used to compare independent groups in terms of categorical variables. The relationship between numerical variables was analyzed by Spearman correlation test when normality was not provided. The following classification was used in the interpretation of Spearman correlation coefficient: r = 0–0.19 (no or negligible relationship), r = 0.20–0.39 (weak relationship), r = 0.40–0.69 (moderate relationship), r = 0.70–0.89 (strong relationship), r = 0.90–1 (very strong relationship) [17].

Linear regression method was used in the modeling established to find the factors predicting global satisfaction with treatment. Factors correlated with global satisfaction were entered into the Enter model. Stepwise variable selection method was used to create a model with only significant independent variables. In the regression analysis, the 95% confidence interval of the risk ratio was also determined. Statistical significance level was accepted as* p* < 0.05.

## Results

### Patients’ characteristics

A total of 110 patients were included [mean (SD) age: 23.79 (4.95) years, 59% male, 46.36% with modulator therapy]. There was no difference in sociodemographic characteristics between the two groups (Table 1).

**Table 1:** Socio-demographic characteristics of the patients

	Total (n = 110)	Modulator therapy group (n = 51)	Non-Modulator therapy group (n = 59)	p*
Gender, n (%)				
Female Male	51 (46.4) 59 (53.6)	27 (52.9) 24 (47.1)	24 (40.7) 35 (59.3)	0.198
Age (years), mean (SD)	23.79 (4.95)	24.03 (5.06)	23.57 (4.88)	0.654
Age of diagnosis (months), Median (min–max)	5 (0–312)	5 (0–276)	4 (0–312)	0.304
Weight (kg), mean (SD)	58.84 (12.95)	59.99 (14.37)	57.85 (11.63)	0.679
BMI (kg/m^2^), mean (SD)	21.16 (3.74)	21.60 (3.81)	20.78 (3.67)	0.181
BMI, n (%)				0.242
< 18.5 kg/m^2^ 18,5.24,9 kg/m^2^ 25–29.9 kg/m^2^ ≥ 30 kg/m^2^	25 (22.7) 69 (62.7) 14 (12.7) 2 (1.8)	10 (19.6) 30 (58.8) 10 (19.6) 1 (2)	15 (25.4) 39 (66.1) 4 (6.8) 1 (1.7)	
Smoking, n (%)	5 (4.5)	1 (1.9)	4 (6.7)	0.370
Use of herbal products, n (%)	9 (8.18)	4 (7.8)	5 (8.4)	1.000

BMI, Body mass index

^*^ Comparison of modulator and non-modulator therapy groups

The number of oral treatments was different between the two groups; no difference was found in other clinical characteristics. (Table 2).

**Table 2:** Clinical characteristics of the patients

	Total (n = 110) Mean (SD)	Modulator therapy group (n = 51) Mean (SD)	Non-Modulator therapy group (n = 59) Mean (SD)	p*
FEV1 (liters)	2.43 ± 1.10	2.45 ± 1.06	2.41 ± 1.14	0.730
FVC (liters)	3.27 ± 1.23	3.34 ± 1.19	3.22 ± 1.27	0.496
FEV1%	66.98 ± 27.08	68.94 ± 27.68	65.28 ± 26.68	0.517
FVC%	77.48 ± 25.90	80.88 ± 26.85	74.54 ± 24.91	0.189
FEV1/FVC	73.16 ± 12.71	72.44 ± 13.84	73.77 ± 11.72	0.721
Number of medications	4.31 ± 2.08	4.50 ± 1.74	4.15 ± 2.34	0.131
Number of oral medications	2.60 ± 1.36	2.80 ± 1.16	2.42 ± 1.51	**0.035**
Number of inhaled medications	1.60 ± 1.21	1.51 ± 1.06	1.67 ± 1.33	0.717
Number of hospitalizations within the last year	0.57 ± 0.92	0.54 ± 0.92	0.59 ± 0.93	0.776
Number of patients hospitalized within the last year, n (%)	40 (36.4)	18 (35.3)	22 (37.3)	0.828
ALT (U/L)	22.84 ± 13.53	24.05 ± 14.51	21.75 ± 12.61	0.384
AST (U/L)	22.93 ± 7.69	23.45 ± 8.8	22.47 ± 6.59	0.519
ALP (U/L)	107.75 ± 54.58	100.62 ± 39.73	114.12 ± 64.76	0.19
GGT (U/L)	24.45 ± 22.98	24.50 ± 18.97	24.40 ± 26.23	0.981
Total bilirubin (mg/dL)	0.72 ± 0.41	0.77 ± 0.43	0.67 ± 0.37	0.250
Albumin (g/dL)	4.24 ± 0.46	4.28 ± 0.36	4.21 ± 0.53	0.346
Creatine kinase (U/L)	83.84 ± 51.50	93.15 ± 43.92	75.51 ± 56.53	0.071
INR	0.99 ± 0.11	0.97 ± 0.07	1.01 ± 0.13	0.104

Bold indicates statistically significant variable

FEV1, First second of forced expiration; FVC, Forced vital capacity; ALT, Alanin aminotransferase; AST, Aspartat aminotransferase; ALP, Alkaline phosphatase; GGT, Gamma-glutamyl transferase; INR, International normalized ratio

Oral medications: Pancreatic enzyme replacement therapy, multivitamin, ursodeoxycholic acid, vitamin A, vitamin E, vitamin D, vitamin D and calcium, azithromycin, proton pump inhibitors, antacids, beta blocker, bisphosphonates, elexacaftor/tezacaftor/ivacaftor, tezacaftor/ivacaftor, ivacaftor

^*^ Comparison of modulator and non-modulator therapy groups

### Comparison of patients using modulator and non-modulator therapies

The only difference between the groups were detected in pancreatic insufficiency and pancreatic enzyme replacement therapy (Table 3).

**Table 3:** CF-related complications and medications of patients

	Total (n = 110), n (%)	Modulator therapy group (n = 51), n (%)	Non-Modulator therapy group (n = 59), n (%)	p*
Pancreatic insufficiency	97 (88.2)	41 (80.4)	56 (94.9)	**0.019**
Metabolic bone disease	35 (31.8)	13 (25.5)	22 (37.3)	0.185
Chronic *Pseudomonas aeuruginosa*	31 (28.2)	18 (35.3)	13 (22)	0.123
CFLD	24 (21.8)	10 (19.6)	14 (23.7)	0.602
ABPA	22 (20)	9 (17.6)	13 (22)	0.566
CFRD	17 (15.5)	9 (17.6)	8 (13.6)	0.554
Pancreatitis	13 (11.8)	3 (5.9)	10 (16.9)	0.073
Gastroesophageal reflux	8 (7.3)	2 (3.9)	6 (10.2)	0.282
Cholelithiasis	5 (4.5)	3 (5.9)	2 (3.4)	0.661
Portal hypertension	4 (3.6)	–	4 (6.8)	0.122
Cholangitis	3 (2.7)	3 (5.9)	–	0.096
Pancreatic enzyme replacement therapy	97 (88.2)	41 (80.4)	56 (94.9)	**0.019**
Dornase alfa	89 (80.9)	41 (80.4)	48 (81.4)	0.898
Multivitamin	67 (60.9)	34 (66.7)	33 (55.9)	0.250
Bronchodilator	41 (37.3)	18 (35.3)	23 (39)	0.690
Ursodeoxycholic acid	23 (20.9)	10 (19.6)	13 (22)	0.755
Inhaled tobramycin	23 (20.9)	14 (27.5)	9 (15.3)	0.117
Inhaled colistin	8 (7.3)	5 (9.8)	3 (5.1)	0.468
Inhaled hypertonic saline	13 (11.8)	3 (5.9)	10 (16.9)	0.075
Inhaled mannitol	8 (7.3)	4 (7.8)	4 (6.8)	1.000
Vitamin A	5 (4.5)	–	5 (8.5)	0.060
Vitamin E	5 (4.5)	1 (2)	4 (6.8)	0.370
Vitamin D	9 (8.2)	2 (3.9)	7 (11.9)	0.172
Vitamin D and Calcium	5 (4.5)	2 (3.9)	3 (5.1)	1.000
Insulin	9 (8.2)	6 (11.8)	3 (5.1)	0.298
Azithromycin	3 (2.7)	1 (2)	2 (3.4)	1.000
Proton pump inhibitors	10 (9.09)	2 (3.9)	8 (13.5)	0.08
Antacids	2 (1.8)	1 (2)	1 (1.7)	1.000
Beta blocker	4 (3.6)	–	4 (6.8)	0.122
Bisphosphonates	2 (1.8)	1 (2)	1 (1.7)	1.000
Elexacaftor/tezacaftor/ivacaftor Tezacaftor/ivacaftor Ivacaftor	40 (36.3) 1 (0.9) 10 (9.1)	40 (78.4) 1 (1.9) 10 (19.7)	–	–
Oxygen therapies	11 (10)	4 (7.8)	7 (11.9)	0.483

Bold indicates statistically significant variable

CFLD, Cystic fibrosis liver disease; ABPA, Allergic bronchopulmonary aspergillozis; CFRD, Cystic fibrosis related diabetes

^*^ Comparison of modulator and non-modulator therapy groups

### Correlation and regression analyses between scales

A positive significant correlation was found between TSQM effectiveness and global satisfaction scores and FEV1%. A negative significant relationship was found between TSQM effectiveness, convenience, global satisfaction scores, and number of inhale medications. While significant correlations were found between TSQM and various domains of CFQ-R, no correlation was found between any domains of TSQM and MARS (Table 4).

**Table 4:** Correlation between treatment satisfaction and clinical outcomes

	Effectiveness		Convenience		Side effects		Global satisfaction	
	r	*p*	r	*p*	r	*p*	r	*p*
FEV1%	0.218*	**0.022**	0.093	0.332	0.078	0.417	0.200*	**0.036**
FVC%	0.294*	**0.002**	0.125	0.194	0.077	0.421	0.272*	**0.004**
FEV1/FVC	− 0.051	0.596	− 0.096	0.320	0.067	0.486	− 0.062	0.522
Number of hospitalizations	− 0.199*	**0.038**	− 0.119	0.216	− 0.052	0.592	− 0.252*	**0.008**
Number of medications	− 0.202*	**0.035**	− 0.322*	**0.001**	0.009	0.929	− 0.137	0.153
Number of oral medications	0.038	0.693	− 0.041	0.674	− 0.133	0.165	0.027	0.779
Number of inhale medications	− 0.378*	** < 0.001**	− 0.371*	** < 0.001**	− 0.087	0.367	− 0.271*	**0.004**
CFQ-R Physical	0.499*	** < 0.001**	0.455*	** < 0.001**	0.058	0.545	0.443*	** < 0.001**
CFQ-R Vitality	0.462*	** < 0.001**	0.438*	** < 0.001**	0.108	0.263	0.454*	** < 0.001**
CFQ-R Treatment burden	0.478*	** < 0.001**	0.611*	** < 0.001**	0.150	0.119	0.435*	** < 0.001**
CFQ-R Emotion	0.317*	**0.001**	0.361*	** < 0.001**	0.074	0.441	0.254*	**0.007**
CFQ-R Eat	0.339*	** < 0.001**	0.331*	** < 0.001**	0.209	0.280	0.269*	**0.004**
CFQ-R Health	0.448*	** < 0.001**	0.459*	** < 0.001**	0.055	0.568	0.414*	** < 0.001**
CFQ-R Social	0.284*	**0.003**	0.251*	**0.008**	−0.079	0.410	0.233*	**0.014**
CFQ-R Body	0.221*	**0.021**	0.200*	**0.036**	0.068	0.481	0.146	0.129
CFQ-R Role	0.410*	** < 0.001**	0.375*	** < 0.001**	0.057	0.556	0.374*	** < 0.001**
CFQ-R Weight	0.183	0.056	0.141	0.141	0.017	0.858	0.189*	**0.048**
CFQ-R Respiratory	0.465*	** < 0.001**	0.474*	** < 0.001**	0.107	0.266	0.459*	** < 0.001**
CFQ-R Digestive	− 0.034	0.723	0.119	0.216	0.070	0.470	− 0.005	0.960
MARS	0.031	0.745	0.091	0.345	− 0.123	0.201	0.051	0.598

Bold indicates statistically significant variable

FEV1, first second of forced expiration; FVC, forced vital capacity; CFQ-R, cystic fibrosis questionnaire-revised; MARS, medication adherence report scale

^*^ Indicates that there is a significant correlation between variables

A significant difference was found between in TSQM domains except for side effects and CFQ-R physical, vitality, treatment burden, nutrition, health, role, weight, respiratory in quality of life (Table 5).

**Table 5:** Comparison of treatment satisfaction of two groups

TSQM	Total (n = 110) Mean (SD)	Modulator therapy (n = 51) Mean (SD)	Non-Modulator therapy group (n = 59) Mean (SD)	p*
Effectiveness	76.18(22.59)	89.64(13.61)	64.12(22.29)	** < 0.001**
Side effects	98.09(6.69)	97.79(6.47)	98.35(6.94)	0.250
Convenience	73.55(20.92)	82.67(15.77)	65.39(21.69)	** < 0.001**
Global Satisfaction	77.83(20.10)	89.63(15.07)	67.28(18.15)	** < 0.001**
CFQ-R Physical	66.15 ± 27.22	72.68 ± 24.06	60.31 ± 28.73	**0.017**
CFQ-R Vitality	61.11 ± 21.91	67.64 ± 20.65	55.26 ± 21.51	**0.001**
CFQ-R Treatment burden	61.11 ± 23.21	67.10 ± 19.99	55.75 ± 24.71	**0.003**
CFQ-R Emotional	72.96 ± 20.76	76.34 ± 20.10	69.94 ± 21.04	0.063
CFQ-R Nutrition	85.28 ± 20.76	89.32 ± 19.99	81.67 ± 20.94	**0.020**
CFQ-R Health	67.69 ± 24.23	73.20 ± 24.15	62.76 ± 23.43	**0.006**
CFQ-R Social	66.25 ± 19.16	68.62 ± 19.74	64.13 ± 18.54	0.174
CFQ-R Body	67.38 ± 32.30	75.81 ± 22.84	59.84 ± 37.48	0.089
CFQ-R Role	80.86 ± 19.41	85.29 ± 16.79	76.90 ± 20.83	**0.013**
CFQ-R Weight	69.75 ± 38.54	81.04 ± 30.00	59.64 ± 42.60	**0.009**
CFQ-R Respiratory	65.48 ± 28.07	77.12 ± 23.45	55.06 ± 27.94	** < 0.001**
CFQ-R Digestion	76.44 ± 24.52	76.90 ± 25.41	76.02 ± 23.92	0.759
MARS	18.54 ± 5.33	18.86 ± 4.61	18.15 ± 5.97	0.954

Bold indicates statistically significant variable

CFQ-R, cystic fibrosis questionnaire-revised; MARS, medication adherence report scale

^*^ Comparison of modulator and non-modulator therapy groups

Patients’ global satisfaction was analyzed by linear regression. Modulator therapy use, FEV1%, CFQ-R respiratory, CFQ-R treatment burden, number of hospitalizations, and number of inhaled medical treatments, which were found to be significantly associated with treatment satisfaction, were entered into the Enter model. The stepwise variable selection method was used to create a model including only significant independent variables. The mean TSQM global satisfaction score of modulator therapy users was found to be approximately 19.733 (95% CI 13.743–25.722; *p* < 0.001) points higher than non-users. When the CFQ-R treatment burden score improved by 1 point, the TSQM global satisfaction score increased by 0.233 points (95% CI 0.105–0.361; *p* < 0.001). In addition, when the number of hospitalizations increased by 1 unit, the TSQM global satisfaction score decreased by 4.751 points (95% CI − 7.899—(− 1.603); *p* < 0.001).

### Drug Related Problems in CF

Each patient’s treatment was evaluated (a median [min–max] of 1 [1–3] times) by the clinical pharmacist. The median number of DRPs per patient was 1, min–max (0–2). A total of 84 DRPs were identified. Of these, 20 (23.80%) were related to treatment effectiveness, and 28 (33.33%) were related to treatment safety. A total of 91 causes were identified for 84 DRPs. It was found that 52 (57.14%) of most DRPs were related to drug selection. When inappropriate drug combinations with other drugs or herbal products were evaluated, interactions were found between modulator therapies and carbamazepine (n = 1), isotretinoin (n = 1), voriconazole (n = 1), clarithromycin (n = 1), colchicine (n = 1), azithromycin (n = 1), risperidone (n = 1), ethinyl estradiol (n = 2), and green tea (n = 2).

In 5 (5.9%) of 84 DRPs, no intervention was performed. The reasons for these problems are the unavailability of mannitol and the patients using the treatment less due to the problem of access to modulator therapy. A total of 141 suggestions were made for 79 DRPs. The most common suggestion was made at the prescriber level (n = 67; 45.89). All 57 physician-level and 12 patient-level suggestions were accepted and implemented. Of the 84 DRPs, 69 (82.1%) were fully resolved. However, 15 (17.9%) issues could not be resolved due to legal and drug access issues. Five patients on modulator therapy reported side effects in the TSQM (pruritus with modulator therapy, cough with tobramycin, flatulence, tachycardia). The classification of DRP is shown in Table 6.

**Table 6:** Classification of drug-related problems (n = 84)

Drug related problems detected (n = 84)	n (%)
**Treatment effectiveness**	**20 (23.80)**
P1.1 No effect of drug treatment despite correct use	–
P1.2 Effect of drug treatment not optimal	4 (4.76)
P1.3 Untreated symptoms or indication	16 (19.04)
**Treatment safety**	**28 (33.33)**
P2.1 Adverse drug event (possibly) occurring	28 (33.33)
**Other**	**36 (42.85)**
P3.1 Unnecessary drug-treatment	5 (5.95)
P3.2 Unclear problem/complaint	31 (36.90)
**Potential or manifest causes (n = 91)**	**n (%)**
**Drug selection**	**52 (57.14)**
C1.1 Inappropriate drug according to guidelines/formulary	3 (3.29)
C1.2 No indication for drug	6 (6.59)
C1.3 Inappropriate combination of drugs, or drugs and herbal medications, or drugs and dietary supplements	14 (15.38)
C1.4 Inappropriate duplication of therapeutic group or active ingredient	–
C1.5 No or incomplete drug treatment in spite of existing indication	26 (28.57)
C1.6 Too many different drugs/active ingredients prescribed for indication	3 (3.29)
**Drug form**	**–**
**Dose selection**	**17 (15.04)**
C3.1 Drug dose too low	4 (3.53)
C3.2 Drug dose of a single active ingredient too high	1 (0.88)
C3.3 Dosage regimen not frequent enough	1 (0.88)
C3.4 Dosage regimen too frequent	6 (6.59)
C3.5 Dose timing instructions wrong, unclear or missing	5 (4.42)
**Treatment duration**	**–**
**Dispensing**	**13 (11.5)**
C5.1 Prescribed drug not available	9 (7.96)
C5.2 Necessary information not provided or incorrect advice provided	3 (2.65)
C5.3 Wrong drug, strength or dosage advised	1 (0.88)
C5.4 Wrong drug or strength dispensed	–
**Drug use process**	**–**
**Patient related**	**9 (7.96)**
C7.1 Patient intentionally uses/takes less drug than prescribed or does not take the drug at all for whatever reason	3 (2.65)
C7.2 Patient uses/takes more drug than prescribed	–
C7.3 Patient abuses drug (unregulated overuse)	–
C7.4 Patient decides to use unnecessary drug	–
C7.5 Patient takes food that interacts	–
C7.6 Patient stores drug inappropriately	1 (0.88)
C7.7 Inappropriate timing or dosing intervals	2 (1.76)
C7.8 Patient unintentionally administers/uses the drug in a wrong way	3 (2.65)
**Patient transfer related**	**–**
**Other**	**–**
**The planned interventions (n = 146)**	**n (%)**
I0.1 No intervention	5 (3.42)
**At prescriber level**	**67 (45.89)**
I1.1 Prescriber informed only	10 (6.84)
I1.2 Prescriber asked for information	–
I1.3 Intervention proposed to prescriber	57 (39.04)
I1.4 Intervention discussed with prescriber	–
**At patient level**	**12 (8.21)**
I2.1 Patient (drug) counselling	8 (5.47)
I2.2 Written information provided (only)	–
I2.3 Patient referred to prescriber	4 (2.73)
I2.4 Spoken to family member/caregiver	–
**At drug level**	**62 (42.46)**
I3.1 Drug changed to	13 (8.90)
I3.2 Dosage changed to	7 (4.79)
I3.3 Formulation changed to	–
I3.4 Instructions for use changed to	12 (8.21)
I3.5 Drug paused or stopped	13 (8.90)
I3.6 Drug started	17 (11.64)
**Other**	**–**
**Acceptance of the Intervention proposals (n = 69)**	**n (%)**
A1.1 Intervention accepted and fully implemented	69 (100)
**Status of the DRP (n = 84)**	**n (%)**
O0.1 Problem status unknown	**–**
O1.1 Problem totally solved	69 (82.1)
O2.1 Problem partially solved	–
O3.1 Problem not solved, lack of cooperation of patient	–
O3.2 Problem not solved, lack of cooperation of prescriber	–
O3.3 Problem not solved, intervention not effective	–
O3.4 No need or possibility to solve problem	15 (17.9)

Bold indicates statistically significant variable

The most common DRPs and interventions are given as examples in Table 7.

**Table 7:** Examples of interventions performed by a clinical pharmacist

DRPs	Example interventions
Patients with chronic *Pseudomonas aeruginosa* and a history of frequent exacerbations	Azithromycin 1 × 500 mg 3 times a week was recommended
Patient moderately sensitive to tobramycin and colistin	The use of alternating antibiotic therapy was recommended
Increased Elexacaftor/tezacaftor/ivacaftor concentration as a result of P-gp inhibition by azithromycin	Azithromycin interruption or toxicity monitoring recommended
Voriconazole- Elexacaftor/tezacaftor/ivacaftor interaction	2 tablets of Elexacaftor/tezacaftor/ivacaftor (100 mg/50 mg/75 mg) were administered in the morning at 3-day intervals and ivacaftor was recommended to be discontinued in the evening
Incorrect use of inhaled antibiotics	Recommended for use after physiotherapy

## Discussion

In this study, treatment satisfaction of 110 CF patients ≥ 18 years of age was assessed, and its relationship with adherence, quality of life, and clinical characteristics was analyzed, and patients with and without modulator therapy were compared.

The key findings of this study were that global satisfaction was influenced by treatment burden and clinical outcomes, including FEV1 and hospitalization. Convenience satisfaction was particularly affected by the number of inhaled medications and decreased with increasing treatment burden. In addition, those using modulator therapy reported higher treatment satisfaction and quality of life. Recommendations were made by the clinical pharmacist to physicians or patients regarding the management of drug-related problems identified in the patient’s treatment. Clinical pharmacist interventions on DRPs were all accepted and implemented by physician and patient-level.

In the study by Greenberg et al., no significant correlation was found between TSQM global satisfaction score for tobramycin treatment and FEV1% [18]. In a study conducted by Wu et al. in chronic obstructive pulmonary disease (COPD) patients, the symptoms of the patients were evaluated with the COPD Assessment Test, and a negative, significant correlation was found with all domains of the TSQM [19]. In our study, we found a significant positive correlation between TSQM effectiveness, global satisfaction scores, and FEV1%. No significant correlation was found between satisfaction with convenience and FEV1%, between satisfaction with side effects and FEV1%. Since CF patients can be prescribed treatment regardless of FEV1%, we may not have detected an association between FEV1% and convenience satisfaction. Since very few patients reported side effects, it is thought that there was no significant relationship between side effects and clinical outcomes.

A significant negative correlation was found between the number of hospitalizations in the last year and TSQM effectiveness satisfaction score, between the number of hospitalizations in the last year and TSQM global satisfaction score. Regression analysis showed that with an increase of 1 unit in the number of hospitalizations, the global satisfaction score decreased by 4.751 points. This shows that patients’ global satisfaction with their treatment is related to their clinical status.

In contrast to the study by Jneid et al. in patients with hypertension, we found a significant negative correlation between the total number of medications and the number of inhaled medications with effectiveness and convenience [20]. When hypertension is not controlled, the addition of antihypertensive therapies leads patients to perceive the treatment as effective, even if the number of medications increases. Most therapies prescribed for CF are aimed at slowing the progression of the disease rather than curing it. Therefore, treatment satisfaction may not improve in CF patients even if the number of medications increases. Inhaled treatments are prescribed especially at low FEV1%, which can affect treatment satisfaction. Therefore, treatment satisfaction can be expected to decrease as the number of inhaled medications increases. No relationship was found between the number of oral medications and treatment satisfaction. This is thought to be due to the prescription of modulator therapies in addition to the routine medications used by patients. Furthermore, those who used modulator therapies reported higher treatment satisfaction than those who did not. In contrast to Regnault et al., we did not find any association between adherence and satisfaction, which may be due to the difference in adherence between modulator therapies and other treatments [6].

In our study, we found a significant relationship between CFQ-R treatment burden and global satisfaction in linear regression. Especially considering that treatment satisfaction is affected by treatment burden, there is a need for recommendations such as dose reduction, inhaled management, and combination therapies that can reduce treatment burden in CF. Since modulator therapies reduce pulmonary exacerbation and improve quality of life, treatment satisfaction and quality of life may have been higher in the group using modulator therapy [21]. However, questionnaires could not be applied to these patients before using modulator therapy.

The role of pharmacists in CF patients has been previously described in the literature [22]. Drug information deficiencies were eliminated, and adherence counseling was performed [23, 24]. In our study, 84 DRPs were detected in a total of 110 patients, 12 of which were potential drug-drug interactions (pDDIs). In terms of DRP management, all recommendations to the physician and patient were accepted and fully implemented, and of those, 82.1% were fully resolved. Patients and physician were also informed about the modulator therapies by the clinical pharmacist. Considering that modulator therapies increase patient survival, it is important that adult CF patients may have various comorbid diseases as they age, and that polypharmacy and pDDIs increase accordingly.

This study has several limitations. The study was conducted in a single center within a limited time. Because modulator therapies are not routinely covered by health insurance, only a limited number of patients could be included in the modulator therapy group. In addition, during the study period, three different physician residents worked in the clinic on a monthly rotation. This situation increased the physicians’ awareness of traditional and modulator therapies with the provision of clinical pharmacy services and limited the clinical pharmacist’s recommendations. Since the study was conducted in the pulmonology department, it limited the recommendations about the medications managed by other departments. As the study consisted of adult patients and most of them had an early age at diagnosis, the patients were generally knowledgeable and aware of their treatment. In addition, treatment satisfaction may be affected by factors such as patients’ family income, living environment and medication preference. This study was not a multicenter study, but according to the 2022 European Cystic Fibrosis Society Patient Registry report, the number of adult CF patients in Turkey was 381 [25]. The study center is a tertiary referral hospital with the highest number of adult CF patients followed in Turkey.

This is the first study in Turkey to compare treatment satisfaction, adherence, and quality of life in adult CF patients with and without modulator therapy. This study is one of the single-center studies with the highest number of CF adult patients in Turkey. Although the detection of DRPs has been previously evaluated in the CF group, the PCNE classification has not been previously used in CF worldwide.

## Conclusion

The multisystemic involvement of CF, its chronic, progressive, and infection-prone nature, and the need for lifelong management increase the number of CF treatments and make treatment management difficult. This study found that treatment satisfaction was particularly affected by the use of modulator therapy and treatment burden. Clinical pharmacists, as part of multidisciplinary management, can reduce treatment burden by patient education, management of DRPs, and contribute to patient health by providing recommendations to improve quality of life. In addition, failures in the delivery of modulator therapies can affect patient quality of life and adherence. Understanding the factors that may influence treatment satisfaction in CF patients is important to ensure patient satisfaction and improve treatment outcomes.

## Data Availability

The data presented in this study are available on request from the corresponding author. The data are not publicly available due to restrictions privacy and ethical.
